# Study of Drug Target Identification and Associated Molecular Mechanisms for the Therapeutic Activity and Hair Follicle Induction of Two Ashwagandha Extracts Having Differential Withanolide Constitutions

**DOI:** 10.1155/2023/9599744

**Published:** 2023-09-30

**Authors:** Franco Cavaleri, Sukalpa Chattopadhyay, Vrushalee Palsule, Pradip Kumar Kar, Ritam Chatterjee

**Affiliations:** ^1^Biologic Pharmamedical Research, 688-2397 King George Blvd, White Rock V4A 7E9, BC, Canada; ^2^Cooch Behar Panchanan Barma University, Panchanan Nagar, Vivekananda Street, Cooch Behar 736101, West Bengal, India

## Abstract

**Background:**

Ashwagandha extracts play a significant role in traditional Indian medicine to help treat a wide range of disorders from amnesia, erectile dysfunction, neurodegenerative and cardiovascular diseases, cancer, stress, anxiety, and many more. Ashwagandha root is enriched with bioactive plant metabolites of which withanolides are the most important ones. The concentration and constitution of withanolides primarily determine ashwagandha's potency and pharmacology. Various factors modulate the withanolide constitution in the plant-derived extracts, rendering inconsistent therapeutic efficacy. Standardisation of the extraction protocol and a better understanding of the pharmacology mechanism of different extracts with varied withanolide constitutions is therefore critical for developing reliable, repeatable, and effective ashwagandha-based treatment.

**Objectives:**

Here, we work toward defining indication mechanisms for two varieties of ashwagandha extract—ASHWITH (ASH-Ext1) and Regenolide (ASH-Ext2)—with different proprietary withanolide proportions.

**Methods:**

ASH-Ext1 was studied for antioxidant signaling modulation using HEK293, HeLa, and A549 cells, and ASH-Ext2 was studied for subcellular drug targets associated with the reactivation and longevity of human hair follicles, using primary human hair follicle dermal papilla cells (HFDPCs).

**Results:**

Study findings support the antioxidant activity and Nrf2 signaling modulation by ASH-Ext1 in various cell models. Of note, ASH-Ext2 was found to increase *β*-catenin and telomerase reverse transcriptase (TERT) protein expression levels in HFDPCs.

**Conclusion:**

The results of drug target modulation show us that the withanolide constitution associated with different extraction protocols influences the pharmacological potential of the extract significantly and points to the value of standardisation not only of total withanolide content but also of internal withanolide proportions.

## 1. Introduction

Ashwagandha (*Withania somnifera*) is one of the most widely used herbs of Ayurveda that belongs to the medicinal plant family Solanaceae. Ayurveda is a traditional system of medicine in India where as much as 80% of the population reports using one form or another of this traditional health care[1]. Ashwagandha has taken a strong position in traditional Indian medicine for as long as 3000 years to demonstrate efficacy in the prevention and therapy of many disorders. These include erectile dysfunction, neurodegenerative and cardiovascular diseases, cancer, and many more and are not limited to amnesia [2]. Ashwagandha is widely considered adaptogenic, meaning it is nontoxic; it can regulate stress and adapt body systems up or down for its overall benefit.

The notion of adaptogenic activity is foreign to allopathic medical models. How this can be weaved into modern medical treatment protocols that may justify the complementary medical potential presents a challenge unless the mechanisms for the pharmacology are better understood, and a standardisation strategy is developed from and for this activity. The multiple bioactive compounds comprising the herb exert properties ranging from anti-inflammatory and antioxidant to immunomodulatory and mood-enhancing properties [2]. Gathering science-based knowledge that unveils which active constituent in the ashwagandha extract is responsible for each of these activities and which pharmacological mechanism provides a strong basis for standardisation, pharmacology validation, and health claims.

For instance, ashwagandha is commonly used in supplemental medicines for children to increase strength [3, 4]; and in adult medicine to support longevity [5, 6], and in general, to help mitigate oxidative stress [7, 8]. The question that has not been convincingly answered today is by which mechanism is “strength” enhanced by which mechanism can we quantifiably measure the potency of this “longevity” claim, and what are the mechanisms by which we can quantify the potential to quench oxidation. Furthermore, which bioactive constituent or which withanolide proportion in the extract reliably delivers these therapeutic effects?

Research has also demonstrated that treatment with ashwagandha can normalize cortisol levels [9] which increase suddenly as a reaction to stress by reducing stress-related anxiety [10, 11]. Which active is responsible and by what mechanism? Ashwagandha is shown to help the user feel more relaxed possibly leading to induced sleep with anxiolytic effects that are comparable to treatment with lorazepam [12, 13]. Supplementation or treatment with the root has shown to double the swimming performance and reduce the stress-related gastrointestinal ulcers in animal research [14, 15]. Ashwagandha's wide range of treatment indications also include improvement of male fertility, treatment of type II diabetes, and improvement of neurodegenerative disorders [15–18]. The indications are many; however, knowing that we are dealing with an herb that can contain in excess of 50 phytochemical actives, it is not entirely surprising. If ashwagandha extracts display variable concentrations and proportions of withanolides and other bioactive constituents such as phenols, flavonoids, tannins, saponins, alkaloids, steroids, terpenoids, and glycosides, the mechanisms and even the active constituents responsible for these therapeutic benefits must be better understood. This herb has also been shown to be effective against inflammation, cancer risks, and can have other positive effects such as antiaging properties, memory enhancement, and regulation of immune functions [2].

The active ingredients in the roots of Ashwagandha are alkaloids, steroidal lactones, and saponins. Withanolides are the more commonly studied constituents [19]. The biologically active metabolites of the herb are primarily the withanolides: a steroidal lactone that has been proven to be the effector against some of the stress-induced and other diseases in humans [20]. These withanolides have even more recently been studied in the context of COVID-19 treatment with promising preliminary antiviral outcomes [21].

A total of 35 withanolides have been extracted from the roots and leaves of the ashwagandha plant to show biological activity. These include withaferin A, withanolide A, withanolide B, and withanolide D and are not limited to withanoside V [2, 20, 22]. A study by Patil et al. [23] shows that the oral bioavailability of withaferin A is almost 1.5 times greater than withanolide A, demonstrating that even defining the final proportions of the withanolides cannot completely predict efficacy in a systemic context based even on the differential bioavailability.

Polypharmacology as opposed to selective pharmacology is a common characteristic of natural extracts, and it should be expected when considering the plethora of actives in a plant extract. Like many botanical or herbal extracts, ashwagandha is comprised of multiple constituents that contribute to this polypharmacology. As much as a “polypharmacology” is factored into the expected treatment outcome in traditional medical applications such as Ayurveda, it can pose as a daunting complexity in the context of modern allopathic medicine principles.

The accepted function of the polypharmacology in the context of wholistic medicinal protocols can fit nicely into the philosophy of wholistic medicine-treatment of the whole system by targeting multiple synergistic subcellular drug targets. In allopathic medicine, however, a similar concept, labelled network pharmacology, speaks a similar language-drug cocktails used to treat complex diseases such as cancers. These cocktails might be pointed at multiple subcellular proteins in a regulatory pathway or multiple cross-talking pathways to deliver a more effective outcome. Despite the application of drug cocktails, mainstream allopathic medicine typically takes an approach to disease treatment that is aligned with selectivity of a drug activity.

Natural botanical extracts have amazing potential as treatment protocols modulating cell signaling and behavior much the same way many allopathic drugs do. Nevertheless, because these extracts are ultimately concoctions of multiple bioactive phytochemicals even if the extract is purified, predictability and repeatability can be a problem. An extract of curcumin that is 97% pure curcumin, for instance, will consist of three curcuminoids of homologous structure, nevertheless, bearing differential molecular features that also afford differential pharmacology [24]. Therefore, if proportions of these actives change the pharmacology changes, the proportions of these constituents will be different from extraction batch to batch based on climate changes, soil type, and agricultural practices that differ in one country or territory over another [25]. Ashwagandha is no different, and the lack of consistency with this herb is likely even a bigger problem due to the higher number of phytoactives. To better predict the biological activity of an ashwagandha extract, the mechanisms by which the purported benefits are delivered must be defined. Concurrently, the responsible active phytochemicals in the final extract could be determined for the targeted mechanism or at the very least a specific withanolide proportion and concentration pinned down and maintained as a standard to lock down a reliable, repeatable, and expected outcome on the bench in clinical trial and also in clinical use as a prescribed treatment [26].

A clinical study by Lopresti et al. [27] shows that consumption of 240 mg of ashwagandha everyday can effectively decrease cortisol-dependent stress level compared to the placebo group. Some animal studies demonstrate that this extract can have positive effects on sex hormone production, sex drive and performance, and even general stamina [4]. However, knowing that one extraction method conveys a different therapeutic outcome over another extraction yield by a differing method, it has us look deeper than just the term “ashwagandha extract” to select the ashwagandha-based treatment [26]. Here, we attempt to set these standards in motion using specifically labelled extractions of the ashwagandha plant extracted by standardised means and studied in the context of specific drug targets associated with antioxidant and redox signaling and other pathways central to redox-related human health and disease. We also journey down a path of research to investigate the potential for an atypically distinct ashwagandha extract that shows a promise with regard to anagen phase induction of hair follicles.

The investigation of mechanisms for various drugs used to modulate autophagy in the context of aging and antiaging which might be applicable to our hair follicle-related objective led us to rapamycin and metformin [28, 29]. Markers and drug targets associated with hair follicle stimulation were also seen in the literature to be positively influenced by these same small-molecule drugs [30]. Subsequently, we found that withaferin A variants show analogous activities to rapamycin [31] justifying the development and study of a withaferin A dominant extraction (as high as 25%) of the ashwagandha root in the context of alopecia. Our research has led to the demonstration of positive influences on key markers of hair growth and general anabolisms such as *β*-catenin and TERT in dermal papilla cells by this unique extract.

## 2. Methods

### 2.1. Cell Lines

HEK293, HeLa, and A549 cells were used to evaluate the efficacy and activity of ASHWITH ashwagandha extract (ASH-Ext1). All three cell lines were kindly provided by Dr. Steven Pelech (Kinexus Bioinformatics, Canada). HEK293 and HeLa cells were grown in Dulbecco's modified Eagle's medium (DMEM) supplemented with 10% FBS and 1% antibiotics. A549 cells were grown in Ham's F12 media supplemented with 10% FBS and 1% antibiotics. DMEM, FBS, and antibiotics were purchased from Sigma, USA, and F12 media were purchased from Corning, USA. The primary human hair follicle dermal papilla cells (HFDPCs) were used to study subcellular targets of Regenolide ashwagandha extract (ASH-Ext2). HFDPCs (# 602-05A) and the complete growth media required to cultivate these cells were purchased from Cell Application, Sigma, USA. Cell cultures were maintained at 37°C in a humidified incubator with 5% CO_2_.

### 2.2. Antioxidant Activity Assay

2,2-Diphenyl-1-picrylhydrazyl (DPPH) assay was performed to determine the antioxidant activity (AA) of ASH-Ext1. DPPH is a stable, free radical that changes its color to light yellow from purple upon reduction by an antioxidant molecule. The % AA of ASH-Ext1 was compared with the commercial ashwagandha extract and gallic acid, the standard antioxidant. In brief, the DPPH stock solution was prepared by dissolving 0.04 mg DPPH (#D9132, Sigma, USA) in 1 ml of ethanol. Two different dilutions (100 and 1000 *μ*g/ml) of each test compound were mixed with DPPH in a 1 : 1 ratio. Sample blank for each dilution of each compound was prepared by mixing sample dilution with a solvent (without DPPH), and the experimental control was prepared by mixing DPPH with a solvent (without sample). The reaction mixtures were vortexed thoroughly and incubated for 30 min in dark at room temperature. The absorbance (Abs) was measured at 517 nm using a spectrophotometer (Helios gamma, Thermo Scientific). The % AA was calculated using the formula % AA = (100 − ((Abs_Sample_ − Abs_Blank_/Abs_Control_) × 100)). Abs_Sample_ = sample dilution + DPPH; Abs_Blank_ = Sample + Solvent; Abs_Control_ = Solvent + DPPH [31, 32]).

### 2.3. MTT Assay

MTT (3-(4,5-dimethylthiazol-2-yl)-2,5-diphenyltetrazolium bromide)-based cell viability assay was done to determine (a) the dose response of ASH-Ext1 and commercial ashwagandha to HEK293 cells, (b) IC50 of hydrogen peroxide (H_2_O_2_) for oxidative stress induction, (c) cytoprotective efficacy of ASH-Ext1 and commercial ashwagandha against oxidative stress, and (d) the dose response of ASH-Ext2 to HFDPCs. In brief, 10 × 10^3^ cells were seeded per well of 96-well plates and were allowed to adhere overnight. On the next day, HEK293 cells were treated with (i) varying concentrations of ASH-Ext1 and commercial ashwagandha (1, 5, 10, 15, 20, and 25 *μ*g/ml) for 24 h, (ii) varying concentrations of H_2_O_2_ (0.2, 0.4, 0.6, 0.8, 1, and 2 mM) for 3 h, and (iii) H_2_O_2_ (0.4 mM) in the absence and presence of ASH-Ext1 (15 *μ*g/ml) or commercial ashwagandha (15 *μ*g/ml) for 24 h. On the other hand, HFDPCs were treated with 0.5, 1, and 2 *μ*g/ml ASH-Ext2 for 24 h. After completion of incubation, drug media were removed and fresh media with 10 *μ*l of 5 mg/ml MTT solution (#M5655, Sigma, USA) were added in each well including control and the blank for 3 h. Afterward, MTT-containing media were removed and DMSO was added to dissolve the formazan crystals. Absorbance was recorded at 540 nm using SpectraMax i3X plate reader (molecular devices). The percentage viability was calculated using the formula % viability = ((Abs_Sample_ − Abs_Blank_/Abs_Control_ − Abs_Blank_) × 100) [33].

### 2.4. ROS Assay

Intracellular ROS level was measured using DCFDA cellular ROS assay kit as per the manufacturer's instructions (Abcam, Canada). ROS fluorescence intensity was detected at Ex/Em = 485/535 nm using SpectraMax i3X plate reader (molecular devices).

### 2.5. HFDPC Clonogenic Activity Assay

HFDPCs are of mesenchymal origin and repertoire of undifferentiated stem cells. Soft-agar colony formation assay [34] was used to determine the clonal expansion capability of HFDPCs treated with ASH-Ext2. In brief, the bottom agar layer (0.5% agar with media) was plated in a 6-well tissue culture plate and allowed to solidify at room temperature. Afterward, the top agar layer (0.3%) containing cells was added to the solidified bottom layer and allowed to settle further for 30 min. Treatment media with (a) DMSO and (b) 1 *μ*g/ml of ASH-Ext2 were added to the control and treatment wells, respectively, twice weekly. Approximately on day 14, colonies were counted manually using an inverted microscope (Nikon Eclipse TS100).

### 2.6. Western Blotting

HEK293, HeLa, and A549 cells were treated with 15 *μ*g/ml of ASH-Ext1 for 24 h, and cell lysates were used to analyze expression levels of Nrf2, HO1, and GPx1. On the other hand, HFDPCs after treatment with ASH-Ext2 at three different concentrations, 0.5, 1, and 2 *μ*g/ml for 24 h, and at 1 *μ*g/ml concentration for 24 and 48 h were subjected to western blot analysis for studying expression levels of *β*-catenin and TERT. *β*-Catenin expression levels were also studied in HFDPCs treated with rapamycin (500 nM) and withaferin A (3 *μ*g/ml) for 24 h. After indicated treatments, cells were harvested using RIPA lysis buffer with 1% protease inhibitor cocktail (Sigma, USA). Total protein was quantified using DC protein assay kit (Bio-Rad), and equal amount of total protein of each sample was separated using polyacrylamide gel electrophoresis (TGX acrylamide kit, Bio-Rad). Proteins were transferred onto nitrocellulose membranes using a wet transfer method. Membranes were blocked with 2.5% BSA blocking buffer for 1 h and probed with primary antibodies such as Nrf2 (2 : 1000, # ab62352), HO1 (2 : 1000, # ab68477), GPx1 (2 : 1000, # ab22604), *β*-catenin (0.2 *μ*g/ml, # ab16051), TERT (1 : 1000, # ab32020)), and GAPDH (1 : 1000, # ab9485) (Abcam, Canada). After overnight incubation at 4°C, membranes were probed with goat anti-rabbit secondary antibody (1 : 10,000) (Sigma, USA) for 1 h at room temperature and were developed with the chemiluminescent agent western HRP substrate (Millipore, Sigma). Membranes were imaged using Fluor-S™ MAX MultiImager (Bio-Rad) and quantified using Quantity One^R^ software [24, 35].

### 2.7. Statistics

Quantitative results were presented as mean ± SD (standard deviation), and Student's *t*-test was used to calculate statistical significance using GraphPad Prism online software. *P* value <0.05 was considered statistically significant.

## 3. Results

### 3.1. Antioxidant Activity and Cytoprotection Efficacy of ASHWITH Ashwagandha (ASH-Ext1)

Antioxidants are free radical scavengers that protect cells by neutralizing these highly reactive molecules. Free radicals are generated in the body endogenously as a by-product of the electron transport chain during mitochondrial respiration as well as exogenously from exposure to toxic chemicals, UV radiation, smoking, and inhalation of pollutant air. In a physiological state, the cellular antioxidant system maintains a fine balance between the generation and detoxification of free radicals to protect the cellular content and is crucial for cell health. However, under oxidative stress conditions, there is free radical accumulation at the cellular and systemic levels which contributes to the development of various pathophysiologies such as atherosclerosis, diabetes, and other chronic conditions such as malignancy. Ashwagandha is well known for its antioxidant and stress-relieving properties for centuries. However, ashwagandha's activity and potency are solely dependent on the total amount of withanolides as well as their unique profile in the extract.

**Figure 1:**
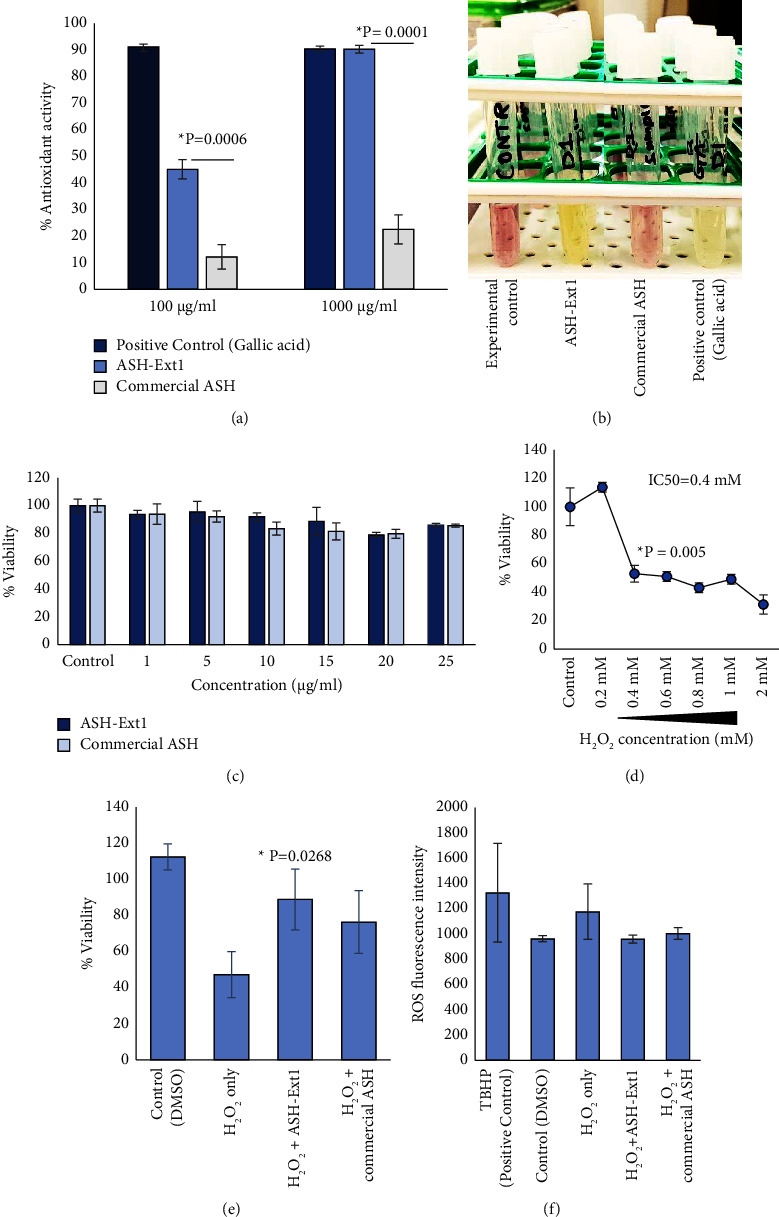
Antioxidant activity and cytoprotection efficacy of ASHWITH ashwagandha (ASH-Ext1) extract: (a) DPPH assay demonstrates the antioxidant activity of ASH-Ext1 and commercial ashwagandha (ASH). Gallic acid was used as the positive control. ASH-Ext1 shows a higher percentage of antioxidant activity as compared to commercial ASH. (b) Pictorial demonstration of the color transformation of DPPH solution in the presence of antioxidant compounds. DPPH free radical upon reduction by antioxidant molecule changes color from purple to yellow. The stronger the reduction, the more intense is the color change. (c) Dose response of ASH-Ext1 and commercial ASH to HEK293 cells. (d) IC50 determination of H_2_O_2_ treatment for inducing oxidative stress in HEK293 cells. (e) Cytoprotection efficacy was measured by MTT assay. HEK293 cells were treated with H_2_O_2_ (0.4 mM) in the absence and presence of ASH-Ext1 or commercial ASH at 15 *μ*g/ml for 24 hr. The result shows a higher percentage of viable cells in the ASH-Ext1 cotreated group in comparison with the H_2_O_2_-treated stressed group (^*∗*^*P* = 0.0268). (f) Reactive oxygen species (ROS) levels were measured in HEK293 cells treated with H_2_O_2_ in the absence and presence of ASH-Ext1 or commercial ASH by DCFDA ROS assay kit. TBHP (tert-butyl hydroperoxide) was used as the positive control. Quantitative data are shown as mean ± SD (standard deviation) of three samples (*N* of 3). ^*∗*^*P* values are considered statistically significant (^*∗*^*P*  <  0.05).

Here we studied the *in vitro* antioxidant activity (AA) and cytoprotection efficacy of ASH-Ext1 against hydrogen peroxide-induced oxidative stress using HEK293 cells. A commercial ashwagandha extract was included for comparison because this extract is known to have similar total withanolide content as ASH-Ext1. The antioxidant activity was measured using *in vitro* DPPH free radical scavenging assay. ASH-Ext1 showed a dose-dependent increment in % AA with significantly higher activity in comparison with the commercial ashwagandha extract (% AA at 100 *μ*g/ml concentration − ASH-Ext1 45.13 ± 3.61 versus commercial ASH 12.24 ± 4.63; ^*∗*^*P* = 0.0006, and at 1000 *μ*g/ml concentration − ASH-Ext1 90.26 ± 1.43 versus commercial ASH 22.55 ± 5.45; ^*∗*^*P* = 0.0001) (Figures 1(a) and 1(b)). To investigate the antistress and cytoprotection efficacy of ASH-Ext1, a hydrogen peroxide (H_2_O_2_)-induced oxidative stress model was established using HEK293 cells. Dose response of HEK293 cells to ASH-Ext1 and commercial ASH was first determined. Both the extracts were found to be noncytotoxic up to the concentration of 25 *μ*g/ml with a cell survival rate above 80% as compared to the control cells (Figure 1(c)). Based on the cell viability data, subsequent experiments were performed using 15 *μ*g/ml concentrations of both extracts. For oxidative stress induction, 0.4 mM concentration of H_2_O_2_ was used which reduced the viability of the cell population by half ^*∗*^*P* = 0.005) (Figure 1(d)). We examined whether ASH-Ext1 or commercial ashwagandha cotreatment protects HEK293 cells against oxidative stress-induced cell death. The percentage cell viability was determined using the MTT assay which showed a reduction in cell survival to 47.22 ± 12.72% after H_2_O_2_ treatment as compared to the control cells, whereas to 88.88 ± 16.83% in H_2_O_2_ and ASH-Ext1 ^*∗*^*P* = 0.0268) and to 76.38 ± 17.34% in H_2_O_2_ and commercial ashwagandha cotreated groups, respectively (Figure 1(e)). Furthermore, intracellular ROS levels were found to be elevated in cells treated with H_2_O_2_ only as compared to DMSO-treated control cells, and subsequent decrease in cells cotreated with ASH-Ext1 or commercial ashwagandha extract (Figure 1(f)). Overall, our findings indicate that ASH-Ext1 has a higher antioxidant activity and cytoprotection efficacy in comparison with the commercial ashwagandha extract and sets the platform for investigating the underlying cellular mechanisms of action of ASH-Ext1.

### 3.2. Modulation of Nrf2 Antioxidant Signaling Pathway by ASH-Ext1 in Various Cell Models

**Figure 2:**
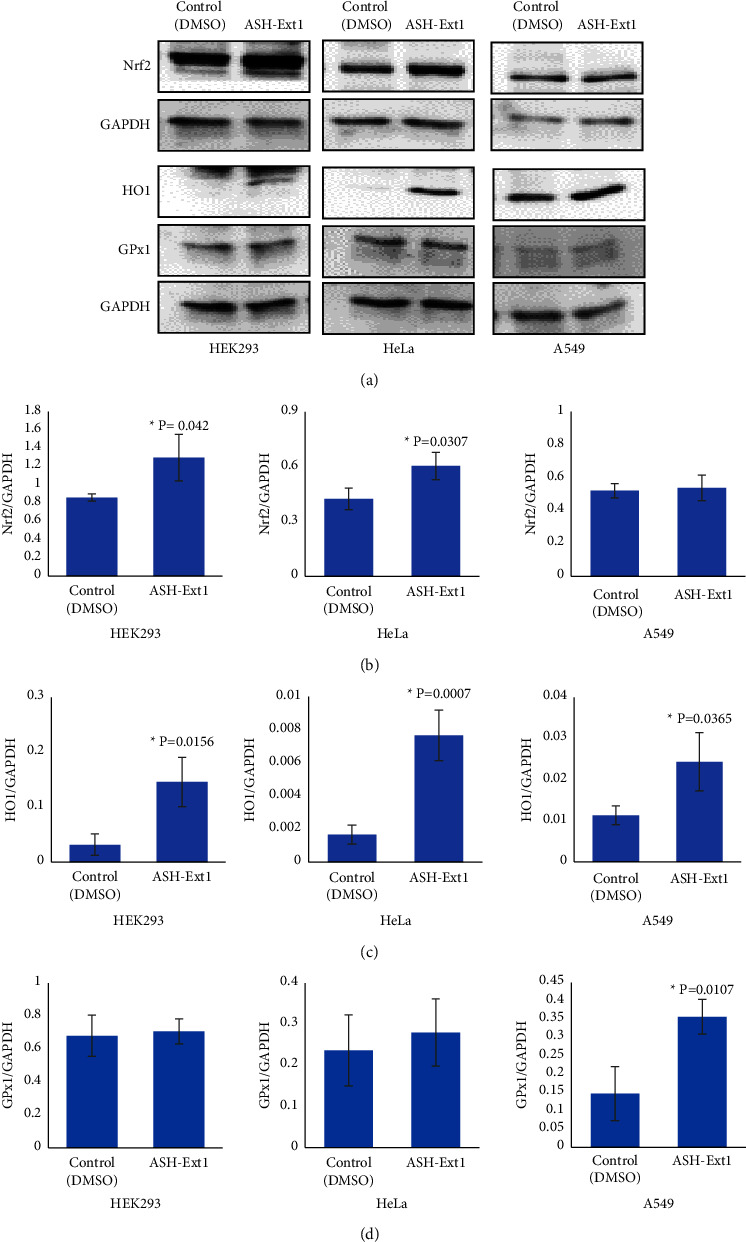
Modulation of antioxidant signaling pathway by ASH-Ext1: (a) HEK293, HeLa, and A549 cells were treated with ASH-Ext1 for 24 h and cell lysates were subjected to western blot analysis to measure expression levels of Nrf2, HO1, and GPx1. The bands are representative of three repeat experiments. Target protein expression was normalized to the samples' loading control GAPDH. Expression levels of (b) Nrf2, (c) HO1, and (d) GPx1 in HEK293, HeLa, and A549 cells. Quantitative data are presented as mean ± SD (standard deviation) of *N* of 3, and ^*∗*^*P* values are considered statistically significant (^*∗*^*P*  <  0.05).

Every organism on this earth is constantly exposed to free radicals and reactive oxidants generated from cellular respiration and environmental toxicants. The primary protective mechanism against oxidative stress is the activation of the antioxidant signaling pathway. Nuclear factor erythroid 2-related factor 2 (Nrf2) is the master transcription factor of this signaling pathway which regulates the expression of an array of cytoprotective and detoxifying genes that counterbalance the adverse effects of these reactive molecules [36, 37]. The antioxidant signaling modulation capability of ASH-Ext1 was studied in HEK293, HeLa, and A549 cells. Cells were treated with 15 *μ*g/ml of ASH-Ext1 for 24 h, followed by protein expression analysis of Nrf2 and its downstream effectors such as HO1 and GPx1. HEK293 cells showed a significant increase in Nrf2 (control 0.86 ± 0.04 versus ASH-Ext1 1.296667 ± 0.254231; ^*∗*^*P* = 0.0423) and HO1 (control 0.031667 ± 0.019553 versus ASH-Ext1 0.146 ± 0.04493328; ^*∗*^*P* = 0.0156) expressions when compared to DMSO-treated control cells. HeLa cells also showed a significant increase in Nrf2 (control 0.423333 ± 0.058595 versus ASH-Ext1 0.603 ± 0.075; ^*∗*^*P* = 0.0307) and HO1 (control 0.001667 ± 0.000577 versus ASH-Ext1 0.007667 ± 0.001528; ^*∗*^*P* = 0.0007) expressions when compared to the control cells. GPx1 expressional alternation was not significant in either cell line. On the other hand, A549 cells showed a moderate increment in Nrf2 expression with a significant increase in HO1 (control 0.011333 ± 0.002309 versus ASH-Ext1 0.02433 ± 0.00709; ^*∗*^*P* = 0.0365) and GPx1 (control 0.14667 ± 0.07371 versus ASH-Ext1 0.3567 ± 0.0473; ^*∗*^*P* = 0.0107) expressions (Figure 2). Overall, study findings indicate that ASH-Ext1 treatment increases the cellular Nrf2 level and differentially modulates the expression of downstream antioxidant molecules.

### 3.3. Augmentation of Hair Growth Promoting Factors in Cultured Primary HFDPCs by Regenolide Ashwagandha Extract (ASH-Ext2) Treatment

**Figure 3:**
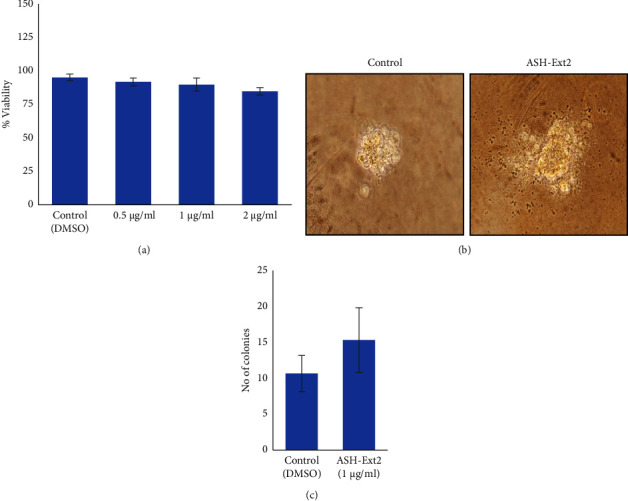
Effect of regenolide ashwagandha (ASH-Ext2) extract on primary HFDPCs: (a) Dose response of ASH-Ext2 to HFDPCs. (b) Microscopic images of HFDPC soft-agar colonies treated with vehicle (DMSO) and ASH-Ext2 at 1 *μ*g/ml (magnification 200x). (c) The result shows improvement in the colony-forming efficacy of HFDPCs treated with ASH-Ext2 when compared to the DMSO-treated control cells. Data are presented as mean ± SD (standard deviation) of *N* of 3.

**Figure 4:**
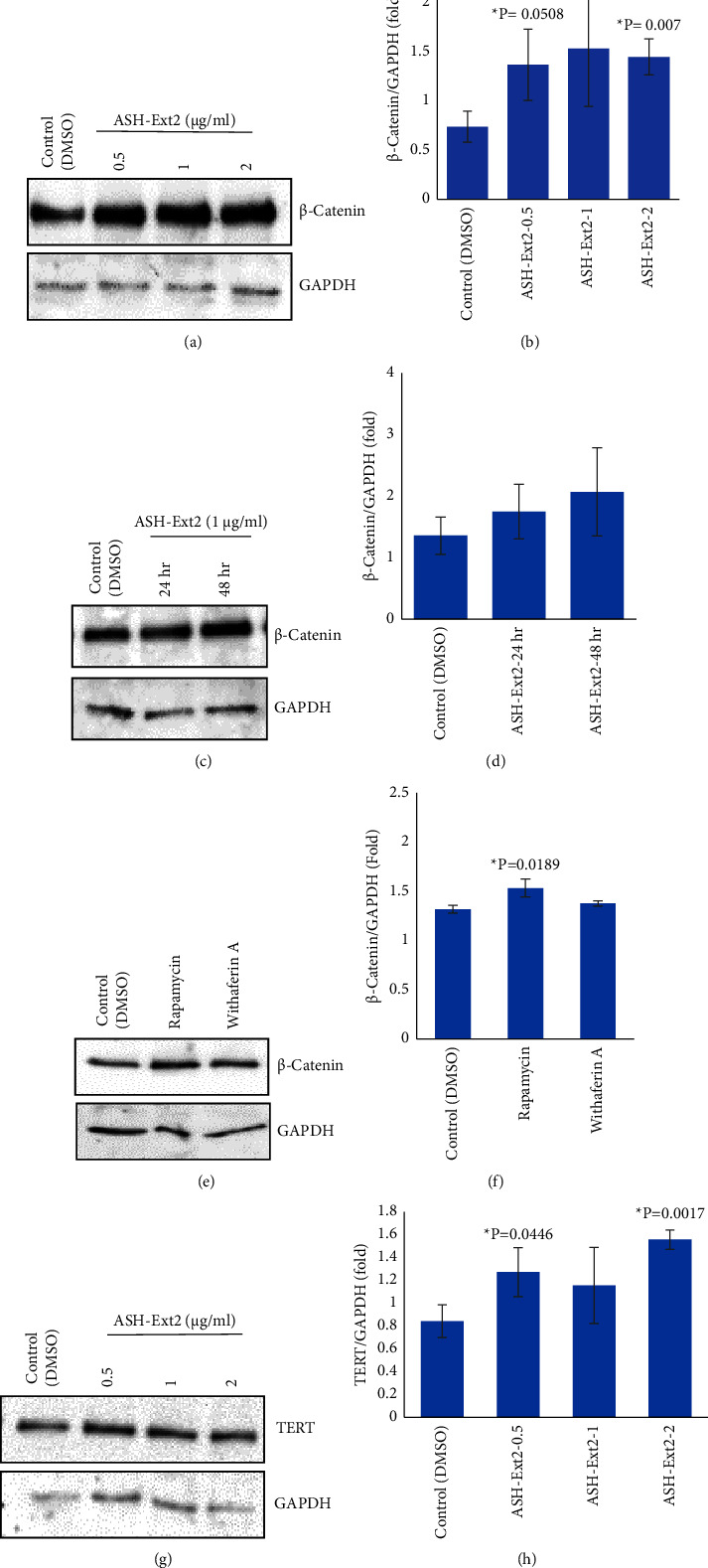
ASH-Ext2 treatment upregulates *β*-catenin and TERT expressions in HFDPCs. HFDPCs were treated with ASH-Ext2 at 0.5, 1, and 2 *μ*g/ml concentrations for 24 h and at 1 *μ*g/ml concentration for 24 and 48 h. Data show (a, b) a dose-dependent and (c, d) a time-dependent increase in *β*-catenin expression in HFDPCs treated with ASH-Ext2. (e, f) *β*-Catenin expression levels in HFDPCs treated with rapamycin and withaferin (A). (g, h) A dose-dependent increase in TERT expression in HFDPCs treated with ASH-Ext2 at 0.5, 1, and 2 *μ*g/ml concentrations for 24 h. Target protein expression was normalized to the samples' loading control GAPDH and presented as fold change with respect to the control. For all data, *N* = 3. ^*∗*^*P* values are statistically significant (^*∗*^*P* ≤ 0.05).

Human hair has multiple functions such as skin protection, preservation of body heat, and sensing and responding to external stimuli. The state of scalp hair can be a factor that influences self-esteem and plays a major role in beauty, heredity, and culture. Even though temporary hair shedding is common in all humans, massive irreversible hair loss or alopecia is often distressing for both men and women and can have a significant impact on psychology and social behavior. Hair follicle dermal papilla cells are specialized cell types of mesenchymal origin that are located at the bottom of hair follicles and are crucial for hair formation and cycling. Here, we aimed at identifying the subcellular targets of Regenolide ashwagandha extract (ASH-Ext2) in primary HFDPCs associated with hair regeneration. *β*-Catenin was considered a primary marker as studies have shown that induction of *β*-catenin activity or expression in the dermal papilla cells leads to the expression of pluripotent factors and ultimately anagen hair cycle induction and folliculogenesis [38–40]. In the present study, the *β*-catenin expression level was studied in primary HFDPCs treated with ASH-Ext2 at varying concentrations (0.5, 1, and 2 *μ*g/ml) as well as for various time points (24 and 48 h). Rapamycin and withaferin A were included in the study for comparison, where rapamycin is known to have hair-inducing potential [41] and withaferin A is a natural mimetic of rapamycin [42]. ASH-Ext2 up to 2 *μ*g/ml concentrations were found noncytotoxic to primary HFDPCs (Figure 3(a)), and at 1 *μ*g/ml concentration, it showed improvement in colony formation when compared to the DMSO-treated control cells (Figures 3(b) and 3(c)). Then, we set out to determine *β*-catenin levels in HFDPCs treated with ASH-Ext2 at 0.5, 1, and 2 *μ*g/ml for 24 h and at 1 *μ*g/ml for 24 and 48 h. A concentration-dependent (0.5 *μ*g/ml ^*∗*^*P* = 0.0508 and 2 *μ*g/ml ^*∗*^*P* = 0.0070) (Figures 4(a) and 4(b)) and a time-dependent (Figures 4(c) and 4(d)) increase in *β*-catenin expressions were observed in ASH-Ext2-treated HFDPCs. An increase in *β*-catenin expression was also observed in the context of rapamycin treatment ^*∗*^*P* = 0.0189) with no significant alteration in withaferin A-treated cells as compared to the control cells (Figures 4(e) and 4(f)). Furthermore, HFDPCs showed a dose-dependent increment in TERT expression, a downstream effector molecule of *β*-catenin signaling pathway (0.5 *μ*g/ml ^*∗*^*P* = 0.0073 and 2 *μ*g/ml ^*∗*^*P* = 0.0017) (Figures 4(g) and 4(h)). Study findings indicate that ASH-Ext2 treatment augments *β*-catenin and TERT expressions in HFDPCs.

## 4. Discussion

Ashwagandha has a reputation in traditional Indian medicine as a versatile Ayurvedic medicinal agent also with applications in sport where its commonly applied to support and advance cardiopulmonary fitness [5, 14], to the treatment of brain disorders [43] including neurodegenerative disorders [44], neurobehavioral disorders such as OCD [45, 46], anxiety disorders [12], memory recall [47], sleep disorders [48], and even the treatment of complex diseases such as cancer [49, 50].

We can show here that many of the drug targets or markers associated with the discussed disease pathologies are, in fact, positively affected by treatment with extracts of ashwagandha. Furthermore, we can demonstrate that various extracts of the same herb perform on the chosen subcellular target proteins in different ways and to different degrees. While regulatory agencies have designed standardisation guidelines for herbal agents that attempt to provide a relative regulatory framework for drug researchers, prescribers, and consumers to reference the protocol for consistency, it all falls grossly short of navigating a consistent outcome.

We can show that yields of the ashwagandha root extracted by variable methodologies produce variable outcomes in the experiments executed here. The range of results is not a surprise when we consider that the ashwagandha extract can have as many as 35 withanolides, 12 alkaloids, and a plethora of other actives in the extract [51]. An ashwagandha extract can be labelled with a withanolide content of 2% for some products and as high as 39% for others. Although, the regulatory framework may mandate label disclosure of the total withanolide content, for example, it does not speak to the internal proportions of the withanolides, 35 of them can shift significantly within the total yield based on the material source and the extraction method. This internal proportion dictates the pharmacological potential of the agent as much as the total of their concentrated sum does. Therefore, it cannot be expected that a 12% withanolide ashwagandha performs the same as the next ashwagandha extract with the same total 12% withanolide content if the plant source and extraction methodology change to alter the internal proportions of the 35 withanolides in that extraction yield.

The results presented here demonstrate that we need a deeper level of standardisation that can allow manufacturers of the naturally extracted medicinal agents such as ashwagandha to extract a consistent outcome that subsequently deliver a consistent pharmacology to consumers. In some cases, such as that of curcumin, where three curcuminoids make up the total extract yield of curcumin, it is easier to manage and hold down a measurable curcuminoid proportion from yield to yield. However, with ashwagandha, the number of withanolides makes it more complicated to pin down consistency. Nevertheless, it is still possible to label extracts with nomenclature that represents a yield that is produced consistently or tested for specific withanolide constitution to ensure that a selective outcome is consistent. In other words, the standardisation process and label claim must include more than just a total extract concentration and must include quantified information about the active withanolides within the total withanolide concentration.

In this report, we present the outcome using two ashwagandha extracts that are produced using two distinctly different processes resulting in very different withanolide proportions. These yields are assigned nomenclature as such: ASH-Ext1 aka ASHWITH ashwagandha and ASH-Ext2 aka Regenolide ashwagandha. ASHWITH ashwagandha, intended for oral use, boasts a withanolide content not less than (NLT) 12%, with a withaferin A content less than 1.5%, while the constitution of Regenolide ashwagandha, designed for topical use, boasts a total 38%–39% withanolide content with as much as 25% of that being withaferin A. The study results are very different and the intended applications are completely diverse, using the same herb to represent different drugs.However, while they each have the same herbal constituents they are arranged in different proportions, to deliver very different pharmacology. With these examples, we also highlight the potential of creating indication-specific profiles of multiple extracts from one herbal agent by modulating the proportions of the active constituents to be more selective for a specific drug target or treatment outcome. ASHWITH™ ashwagandha is poised effectively at countering oxidative stress and has greater efficacy than the other comparable extract tested with it in this study. Here, we show that this targeted activity is not only a function of its direct quenching or antioxidant activity to neutralize the free radical but also a function of its concurrent activity on the Nrf2 signaling pathway. ASHWITH™ ashwagandha is shown to activate transcription through the antioxidant response element by Nrf2 [52]. This signaling pathway is responsible for transcription of downstream intracellular antioxidant proteins HO1 and GPx1. The pathway is also involved in regulation of the NLRP3 inflammasome, a critical drug target for inflammation regulation [52].

On the other hand, we have also studied and reported here on a high withaferin A ashwagandha extract identified as ASH-Ext2 or Regenolide ashwagandha with two immediate objectives: (1) evaluating the potential of this variation to reactivate hair follicles that have become compromised by metabolic or environmental factors and (2) setting the stage for future work with this extract variety on drug targets associated with cancer inhibition.

The higher withaferin A content of Regenolide ashwagandha (ASH-Ext2) extract causes it to qualify more appropriately as a topical agent while we investigate how the oral application in the coming cancer study can be applied to mitigate the toxicity associated with the high withaferin A content. Here, we show that ASH-Ext2 does, in fact, upregulate *β*-catenin effectively in primary HFDPCs. The *β*-catenin activity or expression induces expression of pluripotent factors, anagen hair cycle induction, and folliculogenesis [53]. In experimental work not shown here, we did also investigate the modulatory potential of ASH-Ext1 on HFDPC colony formation (supplemental data) and did not find any reportable outcomes either causing us to void ASH-Ext1 from further investigation in this context. Nevertheless, the activity imparted by ASH-Ext2 on *β*-catenin is something that must be further studied in detail in more ASH-Ext2 constituents in subsequent studies to reveal more about this unknown constituent. We also show a significant upregulation of TERT expression as well as enhanced colony formation by the primary HFDPCs, indicating improved proliferative and replicative activities. These findings indicate a potential for this natural drug to induce anagen phase activity and reactivate hair growth in “hair follicles” that may have reached a stage of senescence or been compromised by other mechanisms leading to alopecia. The modulation of these targets also demonstrates potential for Regenolide ashwagandha extract (ASH-Ext2) to serve as an antiaging and anticancer agent [54] and to deliver therapeutic potential and protection against myocardial ischemia [55], enhancement of myocardial repair [56], and many more.

## 5. Conclusion

Ashwagandha (*Withania somnifera*) is an amazing adaptogenic herbal agent known for millennia to deliver formidable preventive health benefits and therapeutic outcomes. However, standardisation of this and herbal agents, in general, must play a more specific role to guide the regulatory process to indication-specific outcomes.

In this study alone, we investigate the subcellular influences by two distinctly different extractions from ashwagandha. We also study the activities of other known ashwagandha extracts procured from the marketplace. We see a reliable difference in the activity from extract to extract and validation for designing indication-specific variations of these extracts that can be accurately pointed at drug targets with selectivity to potentially treat different diseases or conditions from oxidative stress, premature aging, cancer, and even alopecia. Most patients who use these alternative medicines know they get some level of benefit from the application. However, medical practitioners who prescribe these compounds need to have systems in place that allow them assurances beyond the “ashwagandha extract” description and speak to a properly studied extract that is expected to be processed consistently for a reliable outcome.

## Data Availability

The research data used to support the findings of this study are included within the article.

## Abbreviations

HEK293: Human embryonic kidney cells
HFDPCs: Hair follicle dermal papilla cells
HO1: Heme oxygenase 1
GPx1: Glutathione peroxidase 1
TERT: Telomerase reverse transcriptase
PBS: Phosphate-buffered saline
FBS: Fetal bovine serum.

## Supplementary Materials

Supplementary Materials 1. DPPH free radical scavenging assay for antioxidant activity measurement. Figure description: The antioxidant activities of ASH-Ext1 and ASH-Ext2 were compared using in vitro DPPH free radical scavenging assay. Gallic acid and chlorogenic acid were used as positive controls. Sigma ashwagandha and commercial ashwagandha were used for comparison. Data showed no significant difference in antioxidant activities between ASH-Ext1 and ASH-Ext2, *N* = 3. Ashwagandha was abbreviated as ASH. 2. Colony-forming ability of human hair follicle dermal papilla cells (HFDPC). Figure description: HFDPC cells in soft-agar culture were treated with both ASH-Ext2 and ASH-Ext1 individually and in combination (ASH-Ext2 + ASH-Ext1). Data showed improvement in colony formation in ASH-Ext2 and combination treated groups. No improvement was observed in ASH-Ext1-treated group. *N* = 3.
