# Supplementary material for: Study of Drug Target Identification and Associated Molecular Mechanisms for the Therapeutic Activity and Hair Follicle Induction of Two Ashwagandha Extracts Having Differential Withanolide Constitutions
Source: J Nutr Metab. 2023 Sep 30;2023:9599744. doi: 10.1155/2023/9599744 (PMC10560109; doi:10.1155/2023/9599744)
Supplement: Supplementary Materials — 1. DPPH free radical scavenging assay for antioxidant activity measurement. Figure description: The antioxidant activities of ASH-Ext1 and ASH-Ext2 were compared using in vitro DPPH free radical scavenging assay. Gallic acid and chlorogenic acid were used as positive controls. Sigma ashwagandha and commercial ashwagandha were used for comparison. Data showed no significant difference in antioxidant activities between ASH-Ext1 and ASH-Ext2, N = 3. Ashwagandha was abbreviated as ASH. 2. Colony-forming ability of human hair follicle dermal papilla cells (HFDPC). Figure description: HFDPC cells in soft-agar culture were treated with both ASH-Ext2 and ASH-Ext1 individually and in combination (ASH-Ext2 + ASH-Ext1). Data showed improvement in colony formation in ASH-Ext2 and combination treated groups. No improvement was observed in ASH-Ext1-treated group. N = 3. [file 9599744.f1.docx]

1. **DPPH free radical scavenging assay for antioxidant activity measurement**

**Figure description:** The antioxidant activity of ASH-Ext1 and ASH-Ext2 were compared using in vitro DPPH free radical scavenging assay. Gallic acid and chlorogenic acid were used as positive controls. Sigma ashwagandha and commercial ashwagandha were used for comparison. Data showed no significant difference in antioxidant activity between ASH-Ext1 and ASH-Ext2. N=3; Ashwagandha was abbreviated as ASH.

1. **Colony forming ability of Human hair follicle dermal papilla cells (HFDPC)**

**Figure description:** HFDPC cells in soft-agar culture were treated with both ASH-Ext2 and ASH-Ext1 individually and in combination (ASH-Ext2+ASH-Ext1). Data showed improvement in colony formation in ASH-Ext2, and combination treated groups. No improvement was observed in ASH-Ext1 treated group. N=3
